# Limited Passive Diffusion of Apolipoprotein E Across the Human Blood–CSF Barrier: Evidence From Paired CSF/Serum Quotients

**DOI:** 10.1111/jnc.70585

**Published:** 2026-11-09

**Authors:** Eric Kortmann, Petra Steinacker, Arthur Guthknecht, Lorenzo Barba, Markus Otto

**Affiliations:** ^1^ Department of Neurology University Hospital Halle (Saale), Martin‐Luther University Halle‐Wittenberg Halle (Saale) Germany

## Abstract

Apolipoprotein E (ApoE) is a major genetic risk factor for sporadic Alzheimer's disease and a potential therapeutic target, but the relative contribution of peripheral blood‐derived and intrathecally synthesized ApoE to the CNS remains unclear. A novel electrophoresis‐based capillary Western blot assay was validated to quantify Pan‐ApoE and ApoE4 in paired human CSF and serum samples (*n* = 34), followed by analysis of CSF/serum quotients. Serum ApoE concentrations were ninefold higher than CSF concentrations, with CSF/serum quotients of 0.132 for Pan‐ApoE and 0.191 for ApoE4. Compared with theoretical quotients predicted for proteins entering CSF solely by passive diffusion according to their hydrodynamic radius according to Felgenhauer et al., the observed quotients were 66‐ to 1320‐fold higher for Pan‐ApoE and 96‐ to 1910‐fold higher for ApoE4. Based on this model, passive diffusion was estimated to account for less than 1.5% of CSF ApoE. These estimates are model‐dependent and may vary with ApoE isoform and extent of lipidation, which could potentially be affected by serum fibrin clot formation. The remaining ApoE fraction (> 98.5%) may reflect active transport, intrathecal synthesis or both. Pan‐ApoE and ApoE4 CSF/serum quotients lacked statistically significant association with CSF/serum albumin quotient as a marker of blood‐CSF barrier function. Both quotients also exceeded the values predicted by a theoretical reference curve relating CSF/serum protein quotients to the albumin quotient, adapted from the Reiber model using immunoglobulin G parameters based on the comparable hydrodynamic radii of IgG and ApoE.

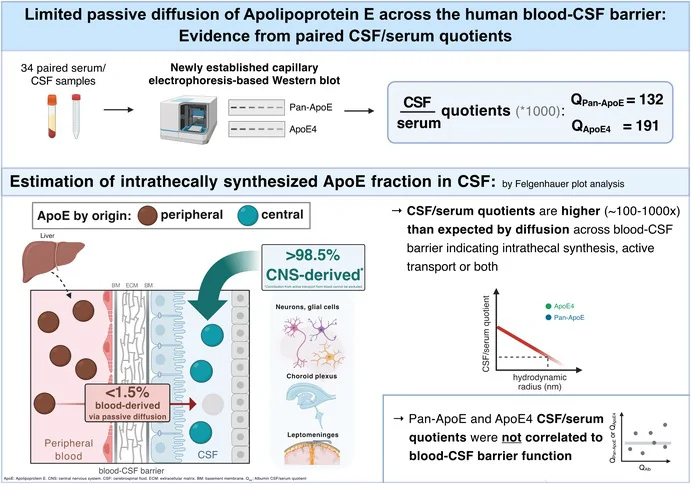

## Graphical abstract

Using a novel capillary Western blot, we quantified Pan‐ApoE and ApoE4 in 34 paired CSF and serum samples. CSF/serum quotient analysis showed that less than 1.5% of CSF ApoE can be explained by passive diffusion across the blood‐CSF barrier, indicating that more than 98.5% originates from the central nervous system through local synthesis and active transport. These estimates are model‐dependent and may vary with ApoE isoform and extent of lipidation. CSF/serum Pan‐ApoE and ApoE4 quotients were not correlated statistically significantly to CSF/serum albumin quotients, as a marker of blood‐CSF barrier function. Given previous experimental and clinical evidence, our findings support a predominantly intrathecal origin of CSF ApoE while not excluding a contribution of active transport across the blood–CSF barrier.

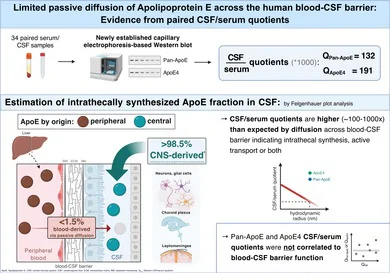

**Abbreviations**
ApoE: Apolipoprotein E
BBB: blood–brain barrier
BCSFB: brain‐cerebrospinal fluid barrier
CI: confidence interval
CLEIA: chemiluminescent enzyme immunoassay
CNS: central nervous system
CSF: cerebrospinal fluid
E2/E3/E4: isoform E2/E3/E4 of Apolipoprotein E
FDR: false discovery rate
IQR: interquartile range
OLS: Ordinary least squares
*Q*_Alb_
: CSF/serum albumin quotient
*Q*_X_
: CSF/serum quotient of a protein “X”
RRID: Research Resource Identifier (see scicrunch.org)

## 1 Introduction

Apolipoprotein E (ApoE) is the most abundant apolipoprotein in the central nervous system (CNS) and exists in three isoforms (ApoE2, ApoE3, and ApoE4). Approximately 80% of Pan‐ApoE is predominantly synthesized in the liver and 20% within the CNS (Kraft et al. 1989; Linton et al. 1991; Moon et al. 2024). Peripheral and central ApoE pools are considered distinct. Following liver transplantation, plasma ApoE isoform expression is shifted toward the donor type, while the CNS ApoE phenotype remains unchanged (Fernández‐Miranda et al. 1997; Kraft et al. 1989; Linton et al. 1991). Experimental studies in mice indicate that peripherally derived ApoE does not appear in the CNS (Liu et al. 2012). However, the extent to which ApoE is exchanged between blood and cerebrospinal fluid (CSF) across the blood‐brain barrier (BBB) or blood‐CSF barrier (BCSFB) in humans remains poorly understood. These two barriers are often used interchangeably, yet they are functionally and structurally distinct entities: the BBB constitutes a highly selective barrier formed primarily by the neurovascular unit, whereas the more permeable BCSFB comprises the choroid plexus epithelium with fenestrated capillaries and subarachnoid epithelial cells lining the CSF space (Abbott et al. 2006; Castro Dias et al. 2019; Engelhardt and Sorokin 2009). A precise distinction between these two interfaces is therefore essential when interpreting ApoE exchange data.

BBB dysfunction is typically associated with structural impairment resulting in loss of size‐dependent permeability for macromolecules and blood cells (Engelhardt and Sorokin 2009). In contrast, dysfunction of the BCSFB does not involve changes in the physical structure of the barrier as might be implied by misleading terms of barrier “impairment” or “leakage” (Reiber 1994). Rather, BCSFB dysfunction is functionally defined as increased concentrations of blood‐derived proteins in CSF, which occur as a consequence of diminished elimination due to CSF flow reduction (Reiber 1994, 2001, 2016, 2021).

Therefore, the total concentration of proteins in CSF reflects a dynamic balance between blood‐derived proteins entering the CSF via passive transfer across the BCSFB, brain‐derived proteins produced by neurons, glial cells, and choroid plexus, and proteins secreted by leptomeningeal cells into the CSF, and their subsequent drainage through the arachnoid granulations into the venous system or via the glymphatic route into the lymphatic system (Benveniste et al. 2017; Deisenhammer et al. 2015; Proulx 2021; Reiber 1994). This relationship is described by Reiber's hyperbolic function, which accounts for the nonlinear dependency of CSF/serum quotients of blood‐derived proteins on albumin quotient (*Q*_Alb_), which is a reciprocal measure of CSF flow and thus the aforementioned functionally defined BCSFB function (Reiber 1994, 2016).

For ApoE, most previous estimates of the CSF/serum quotient and the intrathecal fraction were based on molecular weight considerations and ApoE CSF and serum concentration measurements derived from different laboratory methods from the 1990s (Pitas et al. 1987; Thomas 1998). These approaches may be inaccurate for proteins with a relatively large hydrodynamic radius, such as ApoE (Felgenhauer 1974). Carlsson et al. reported serum ApoE concentrations approximately 12‐fold higher than CSF concentrations, based on ELISA measurements of paired serum and CSF samples (Carlsson et al. 1991). In contrast to Pan‐ApoE, CSF/serum quotients for ApoE4 have not previously been determined, and the fraction attributable to passive diffusion across the BCSFB has therefore not been estimated to our knowledge after extensive literature research.

The ApoE isoforms differ by single amino acid substitutions and glycosylation, resulting in distinct structural and functional properties (Deane et al. 2008; Fernández‐Calle et al. 2022; Moon et al. 2024; Troutwine et al. 2022). For example, the ApoE4 isoform is associated with decreased amyloid clearance and an increased risk of sporadic late‐onset Alzheimer's disease (AD), whereas ApoE2 confers relative protection (Corder et al. 1993; William Rebeck et al. 1993). Recent evidence suggests that *APOE* risk alleles may account for more than 70% of the AD burden in European cohorts (Williams et al. 2026). Accordingly, interest in ApoE‐targeting therapies has grown, including experimental isoform‐switching from deleterious ApoE4 to protective ApoE2 or a Phase 2 clinical trial of bumetanide, a precise understanding of the origin and compartmentalization of ApoE isoforms in CSF is essential for developing future therapeutics and anticipating potential adverse effects along with their underlying pathophysiology (Cummings et al. 2025; Golden et al. 2025).

In this study, we examined the relationship between serum and CSF ApoE by analyzing paired samples using an electrophoresis‐based capillary Western blot after validating the method. To our knowledge after extensive literature review, this is the first study to determine CSF/serum quotients of ApoE and evaluate the relationship between serum and CSF ApoE concentrations using an electrophoresis‐based capillary Western blot. By quantifying CSF/serum quotients of Pan‐ApoE (*Q*_Pan‐ApoE_) and ApoE4 (*Q*_ApoE4_) isoform, we assessed the contribution of passive diffusion of Pan‐ApoE and ApoE4 from blood to CSF according to Felgenhauer (Felgenhauer 1974) and examined the relationship of both analytes on BCSFB function. Moreover, we evaluated the association between serum and CSF ApoE levels and compared CSF/serum quotients with a theoretical Reiber reference curve according to the hydrodynamic properties of ApoE (Reiber 1994). Separate analysis of ApoE4 allowed us to investigate isoform‐specific patterns of blood‐to‐CSF transfer and its relationship with BCSFB function.

We hypothesize that passive diffusion of ApoE across the BCSFB contributes only minimally to CSF Pan‐ApoE and ApoE4 concentrations, and that CSF levels are therefore only weakly associated with serum ApoE concentrations and BCSFB function. However, the relative contributions of intrathecal synthesis and active transport could not be distinguished in the present study, and the estimated fractions should be interpreted as model‐based approximations (Felgenhauer 1974; Reiber 1994).

## 2 Methods

### 2.1 Ethics Approval and Consent to Participate

The study was approved by Martin Luther University's Halle‐Wittenberg Ethics Committee (2021‐101) and conducted according to the latest version of the Declaration of Helsinki. All participants gave informed consent to participate in the study before taking part. This observational human‐subjects study was not preregistered.

### 2.2 Patient Selection

CSF samples obtained by lumbar puncture and paired serum samples from 34 individuals were collected at the Department of Neurology, Martin Luther University Halle‐Wittenberg. Patient characteristics are shown in Table 1 (see Supplementary material, Table 1). Patients were selected based on the availability of paired CSF and serum samples and known *APOE* genotype or the presence of BCSFB dysfunction to ensure adequate representation across a broad range of CSF/serum albumin quotient (*Q*_Alb_) values. No formal a priori sample size calculation or power analysis was performed, as this was an exploratory study. The sample size was determined by the availability of paired CSF and serum samples that met the predefined inclusion criteria during the study period. Patients were selected to ensure representation of both normal and impaired BCSFB function as well as *APOE ε4* carriers. To examine the relationship between CSF ApoE levels and BCSFB function, 14 patients with BCSFB dysfunction, as indicated by an increased Q_Alb_ above the age‐specific cut‐off, and 20 patients with normal BCSFB function were included. Of the 34 patients included in the study, 18 had a diagnosis of AD and 23 carried an *APOE ε4* genotype. Patients with evidence of intrathecal IgG synthesis were excluded to avoid confounding by intrathecal immunoglobulin production. No samples were excluded due to insufficient CSF volume or blood contamination from traumatic lumbar puncture. Detailed patient diagnoses are provided in the supplementary material (Supplementary material, Table 2). Analysts were not blinded to diagnosis, *APOE* genotype, or BCSFB status during quantification, as blinding was not applicable to this study.

### 2.3 Sample Handling

CSF samples of varying volumina (mean: 10.1 mL, SD: 1.3 mL, range: 7–13 mL) were obtained by lumbar puncture followed by blood serum collection using S‐Monovette Serum CAT (02.1063.001, SARSTEDT AG & Co. KG, Nümbrecht, Germany) under hygiene precautions. CSF was collected once from each patient. Potential blood contamination was assessed based on the CSF erythrocyte count obtained during routine CSF analysis. Erythrocyte counts ranged from 0 to 31 cells/μL (mean (SD): 3 (7) erythrocytes/μL). Both matrices were centrifuged at room temperature (CSF: 1200 g, 10 min, serum: 2000 g, 10 min), aliquoted, and stored at −80°C within 2 h until analysis.

### 2.4 Principle of Electrophoresis‐Based Capillary Western Blot

The 34 paired CSF and serum samples were analyzed by capillary‐based electrophoresis and Western blot using the PeggySue instrument (RRID:SCR_020406, Protein Simple, Bio‐Techne) and the 12–230 kDa Separation Kit (cat. no. SWPS‐ST01‐1L, Bio‐Techne). Each capillary is automatically filled with stacking and separation matrices and the respective sample by the instrument. Proteins in the sample are then size‐separated and immobilized on the capillary wall by ultraviolet light exposure. After incubation of each sample with anti‐ApoE4 and anti‐Pan‐ApoE primary antibodies in distinct capillaries, the chemiluminescence signal generated by the horseradish‐peroxidase (HRP) conjugated secondary antibody is detected by the instrument and the signal quantitatively analyzed and presented in arbitrary units using the compatible Compass Software.

### 2.5 Preparation for Electrophoresis‐Based Capillary Western Blot

After diluting each sample to concentrations within the linear range of the detection system (CSF: 25–30 mg/L total protein concentration, serum: 1:400) with 0,1X Sample Buffer (no RRID found, cat. no. 042‐195, Bio‐Techne) and mixing with reconstituted fluorescent 5X master mix (12–230 kDa) at a 4:1 ratio according to manufacturer's guidelines, the mixture was heated (95°C, 5 min), vortexed and stored on ice until pipetted to the 384‐well assay plate together with the molecular weight ladder, blocking reagent (antibody diluent), primary and secondary antibodies, luminol/peroxide, as well as the stacking and separation matrix according to manufacturer's guidelines and plate layout. The sample diluent (“Sample Buffer”, no RRID found, cat. no. 042‐195, Bio‐Techne) contains 200 mM Bicine, pH 7.6, 6% CHAPS, 10% SDS according to the manufacturer.

The following assay protocol was defined in the Compass Software: Load time of separation matrix: 150 s, stacking matrix 15 s, sample 9 s. Separation: 40 min, 250 V. Standard exposure: 5 s. EE immobilization time: 250 s. Matrix removal: 230 s, 3 matrix washes. Antibody diluent blocking: 23 min, 3 washes. Primary and secondary antibody incubation: 30 min, followed by 2 washes. Detection Profile: High Dynamic Range 4.0.

### 2.6 ApoE‐Isoform Specificity and Linearity of Pan‐ApoE and ApoE4 Antibodies

Each CSF and serum sample was incubated with a monoclonal ApoE4 isoform specific antibody (no RRID found, cat. no. 39327, CellSignaling, host: rabbit) and a polyclonal anti‐Pan‐ApoE antibody (RRID:AB_2878525, cat. no. 18254‐1‐AP, proteintech, host: rabbit) that recognizes every ApoE isoform (E2, E3, E4), both applied in a 1:10 dilution. A HRP‐conjugated goat anti‐rabbit‐IgG (RRID:AB_2534782, cat. no. A16110, invitrogen) secondary antibody was used at a 1:20 dilution to detect the primary antibodies.

The isoform specificity of the anti‐Pan‐ApoE (Figure 1A) and anti‐ApoE4 (Figure 1B) antibodies was confirmed by testing serum and CSF samples from patients representing all *APOE* genotypes, with the exception of the *APOE ε2*/*ε2* genotype, for which only serum was available. Peaks were identified manually using the electropherogram (Figure 1C).

**FIGURE 1:**
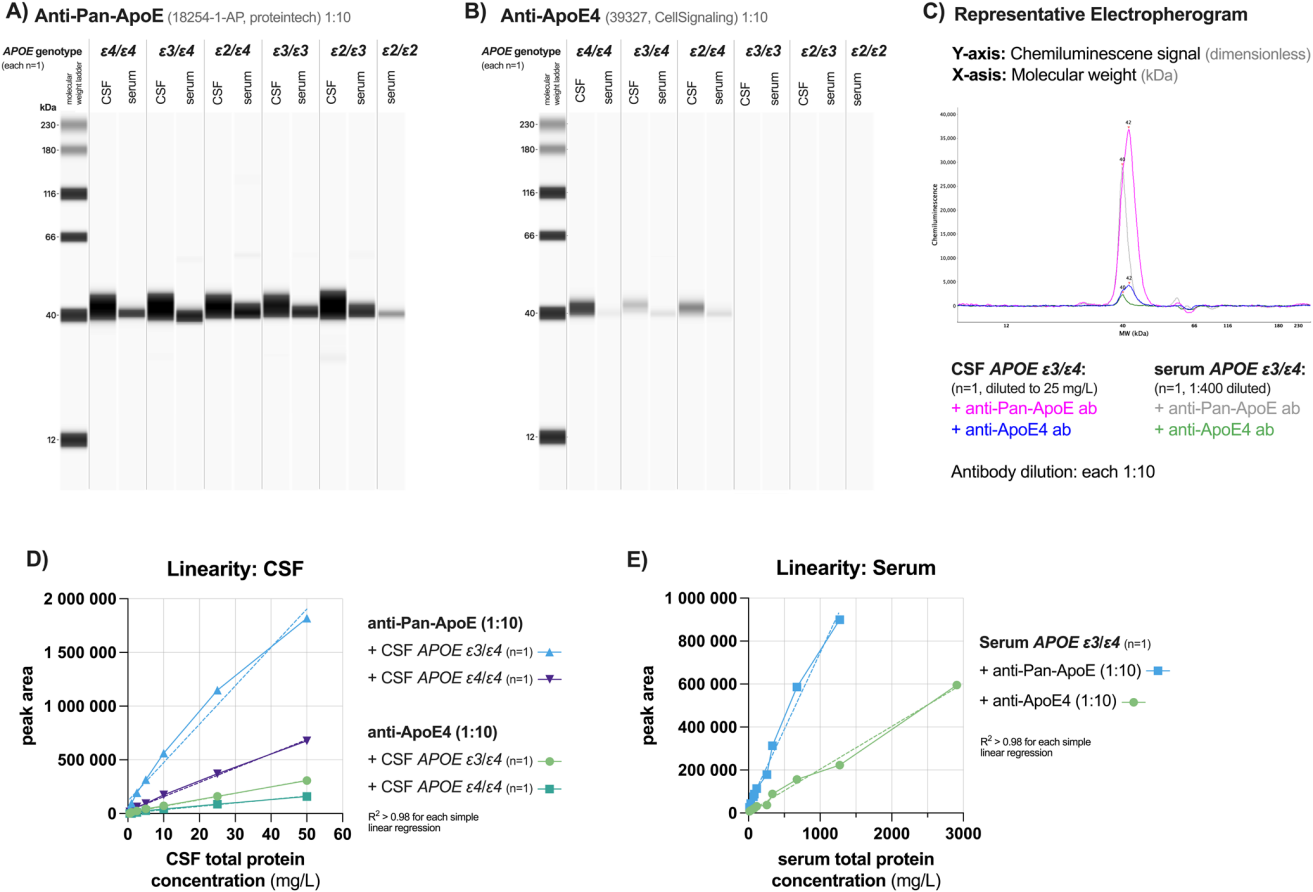
Validation of the ApoE4 and Pan‐ApoE electrophoresis‐based capillary Western blot. (A, B) Western blots of serum (1:400 dilution) and cerebrospinal fluid (CSF) (diluted to 25 mg/L total protein concentration) probed with the anti‐Pan‐ApoE antibody (A) and anti‐ApoE4 antibody (B) from patients with *APOE* genotypes *ε4*/*ε4*, *ε3*/*ε4*, *ε2*/*ε4*, *ε3*/*ε3*, *ε2*/*ε3*, and *ε2*/*ε2* (each *n* = 1). For the *APOE ε2*/*ε2* genotype only serum was available. Bands at 40 kDa in both serum and CSF are observed for every ApoE isoform with the Pan‐ApoE antibody, whereas the anti‐ApoE4 antibody detects 40 kDa bands in both matrices solely in *APOE ε4* genotypes. (C) Representative electropherogram illustrating chemiluminescence signal intensity as a function of molecular weight for CSF (*APOE ε3*/*ε4*, diluted to 25 mg/L) and serum (*APOE ε3*/*ε4*, 1:400 dilution) samples detected with anti‐Pan‐ApoE and anti‐ApoE4 antibodies. (D) Linearity of the Western blot signal (peak area) increase in CSF was assessed using CSF samples from patients with *APOE ε3*/*ε4* and *APOE ε4*/*ε4* genotypes in serial dilutions. (E) Linearity of the peak area increase in serum was evaluated by testing a serum sample from a patient with an *APOE ε3*/*ε4* genotype in serial dilutions.

Serial dilutions of CSF (400.0–0.5 mg/L) and serum (2900.0–15.0 mg/L, corresponding to dilutions from 1:20 to 1:4000) were analyzed to assess the linearity of the Western blot signal, defined as an *R* ^2^ > 0.98 in simple linear regression (Figure 1D,E). The anti‐ApoE4 antibody exhibited a linear increase in the area under the peak across the entire range of serum dilutions tested, whereas the anti‐Pan‐ApoE antibody showed linearity only between 15 and 1270 mg/L (corresponding to serum dilutions of 1:4000–1:50). For CSF samples, both antibodies demonstrated a linear increase in peak area within a total protein concentration range of 0.5–50.0 mg/L.

### 2.7 Electrophoresis‐Based Capillary Western Blot Data Analysis and CSF/Serum Quotient Calculation

Compass Software (Bio‐Techne) was used to quantitatively analyze the data gained by capillary‐based electrophoresis and Western blot. After checking the molecular weight standards and registration, peaks at the expected molecular weight of ApoE (34–40 kDa) were identified using the electropherogram (Figure 1C). The software automatically calculates the area under the peak (gaussian fit) for each peak. The quotient of the ApoE peak area in CSF and serum for each sample pair was calculated by dividing the peak area of CSF by that of serum (*Q*_Pan‐ApoE_ = peak area ApoE_CSF_/peak area ApoE_serum_), after correcting each peak area for the corresponding sample dilution. The same principle was applied for the data gained by the anti‐ApoE4 antibody (*Q*_ApoE4_ = peak area ApoE4_CSF_/peak area ApoE4_serum_). Similar to the albumin quotient, the ApoE (CSF/serum) quotient was multiplied by 1000 to obtain values greater than zero.

### 2.8 Theoretical ApoE Reibergram to Assess the Origin of ApoE

Reiber graphs were originally empirically established for Immunoglobulins IgG, IgA, and IgM, describing the relationship between the respective CSF/serum protein quotients and the albumin quotient (*Q*_Alb_) as a measure of BCSFB function (Reiber 1994). This relationship follows a hyperbolic function reflecting the nonlinear increase of protein CSF/serum quotients with increasing *Q*_Alb_. For ApoE, a theoretical hyperbolic reference curve was estimated by using the Reiber model. The hydrodynamic radius of ApoE (ranging from 4.8 to 8.7 nm, depending on ApoE isoform and lipidation) is close to the hydrodynamic radius of IgA and IgG (Hubin et al. 2019; Raulin et al. 2019; Reiber 1994). Therefore, the empirically determined parameters from IgG (*a*/*b* = 0.65, *b* = 8, *c* = 1.4) were applied to estimate a theoretical Reiber curve for ApoE (Equation 1), as has previously been done for other proteins (Padilla‐Docal et al. 2009; Reiber 1994; Reiber et al. 2012). This approach allows an approximate estimation of the expected CSF/serum ApoE ratio under conditions of passive diffusion of ApoE across the BCSFB, in the absence of intrathecal synthesis or active transport.

(1) $$Q_{\text{ApoE}}=\frac{a}{b}\cdot \sqrt{Q_{\mathrm{Alb}}^{2}+b^{2}}-c$$

Although this relationship is mathematically hyperbolic, within the observed range of *Q*_Alb_ values it is nearly linear. Therefore, simple linear regression of the observed *Q*_Pan‐ApoE_ as well as *Q*_ApoE4_ and *Q*_Alb_ was performed and compared to the theoretical Reiber curve.

### 2.9 *APOE* Genotype Determination by Allele Specific Quantitative Real‐Time PCR

From 27 out of a total of 34 patients, the *APOE* genotype was determined by allele‐specific quantitative real‐time Polymerase Chain Reaction (qPCR) using the DiaPlex Apolipoprotein E (ApoE) Genotyping Kit (cat. no SQH01‐K100, SolGent Co. Ltd. Yuseong‐gu, Daejeon, Korea) according to the manufacturer's guidelines. A QuantStudio 3 (RRID:SCR_018712, Thermo Fisher Scientific, Waltham, MA, USA) was used as a qPCR instrument. Extracted DNA as sample material was obtained from serum or plasma using the NucleoSpin cfDNA XS Kit (no RRID found, cat. no. 740900.50, MACHERY‐NAGEL GmbH & Co. KG, Düren, Germany).

### 2.10 Lumipulse ApoE4 and Pan‐ApoE Assay

Serum samples from 33 and CSF samples from 31 out of 34 patients were analyzed for ApoE4 and Pan‐ApoE concentrations using the chemiluminescent enzyme immunoassays (CLEIA) Lumipulse G ApoE4 and Pan‐ApoE (no RRID found, cat. no. 81453 and 81449, Fujirebio Europe N.V., Gent, Belgium; Research Use Only) on the Lumipulse G600II (RRID:SCR_023515) platform. Calibration was performed with Lumipulse Pan‐ApoE and ApoE4 Calibrators (no RRID found, cat. no. 81454 and 81450, Fujirebio Europe N.V., Gent, Belgium) according to the manufacturer's instructions. For quality control, Lumipulse G ApoE Controls (no RRID found, cat. no. 81452, Fujirebio Europe N.V., Gent, Belgium) at two concentration levels for both Pan‐ApoE and ApoE4 were measured and found to be within the manufacturer's specified range. Subsequently, the serum and CSF samples were analyzed according to the manufacturer's protocol on separate days.

### 2.11 Association Between ApoE Western Blot and Lumipulse ApoE Measurements

To assess whether the Western blot signal intensities in arbitrary units (peak area) reliably reflect absolute protein concentrations, we examined the association between the Western blot results and the corresponding absolute concentrations measured by the Lumipulse CLEIA in both serum and CSF. Due to insufficient sample volume for Lumipulse assays in some serum and CSF samples, the analyses were performed on the subset of samples with sufficient volume for both methods. Both serum Pan‐ApoE and ApoE4 Western blot peak areas showed a strong correlation (serum Pan‐ApoE *r*_s_: 0.6110, 95%‐CI: 0.3294–0.7928, *p* = 0.0002, FDR‐adjusted *p* = 0.0013, *n* = 33, serum ApoE4 *r*_*s*_: 0.5762, 95%‐CI: 0.2130–0.7794, *p* = 0.0032, FDR‐adjusted *p* = 0.0104, *n* = 24) with the respective Lumipulse‐determined absolute concentrations (Figure 2A,B). CSF ApoE4 Lumipulse concentrations and Western blot signals were strongly correlated (CSF ApoE4 *r*_s_: 0.6810, 95%‐CI: 0.3523–0.8601, *p* = 0.0005, FDR‐adjusted *p* = 0.0022, *n* = 22). Pan‐ApoE showed a marginally weaker association between the two methods which was statistically non‐significant after correcting for multiple testing (CSF Pan‐ApoE *r*_s_: 0.3921, 95%‐CI: 0.0329–0.6616, *p* = 0.0291, FDR‐adjusted *p* = 0.0631, *n* = 31) (Figure 2C,D).

**FIGURE 2:**
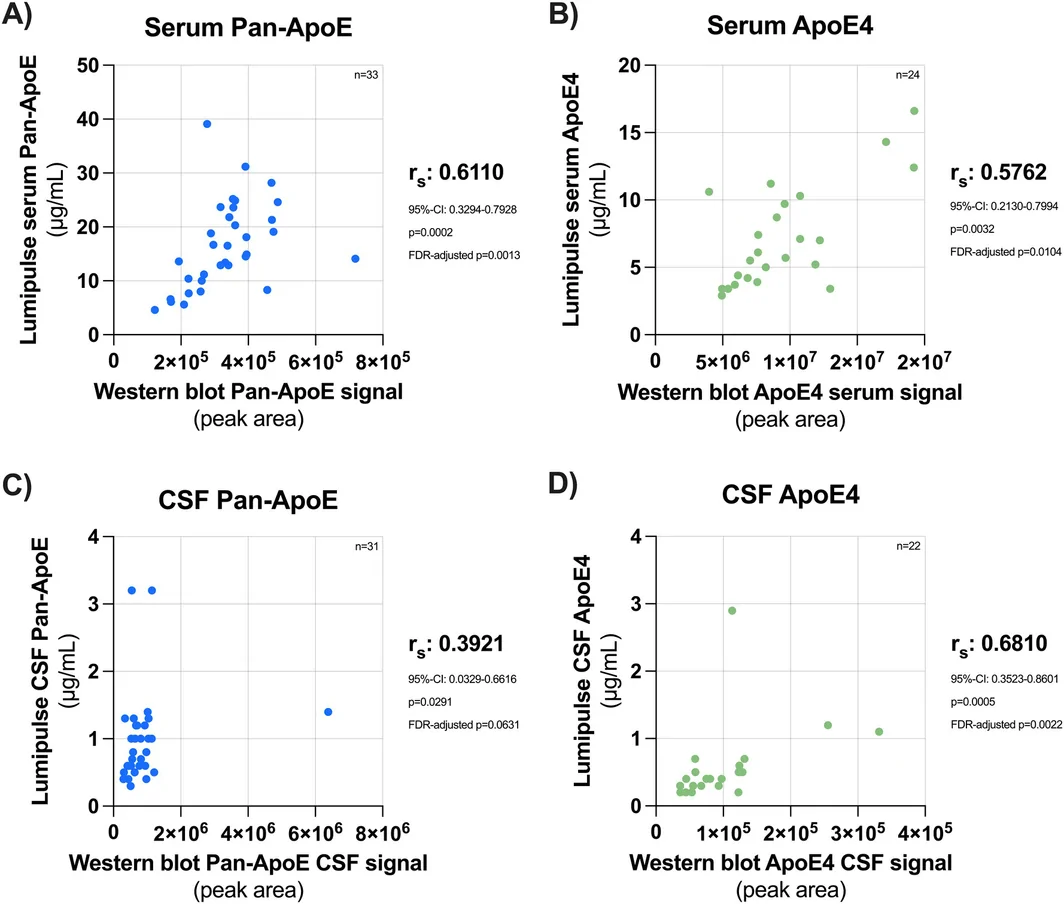
Association between serum Western blot signal intensity and absolute concentration determined by chemiluminescent enzyme immunoassay of Pan‐ApoE and ApoE4. Plots show data from patients with available Western blot and Lumipulse measurements in serum (A, B) and CSF (C, D) for Pan‐ApoE (serum: *n* = 33, CSF: *n* = 31) and ApoE4 (serum: *n* = 24, CSF: *n* = 22).

### 2.12 Statistical Analysis

Statistical analysis was done and plots were created by using GraphPad Prism version 10.3.1 (GraphPad Software, San Diego, CA, USA). The Reibergram (Figure 4A) was created by using CSF Research Tool/Reibergrams Protein Statistic version 4.63 (Albaum IT‐Solutions, Werner Albaum with Prof. Hansotto Reiber, Göttingen, Germany).

**FIGURE 3:**
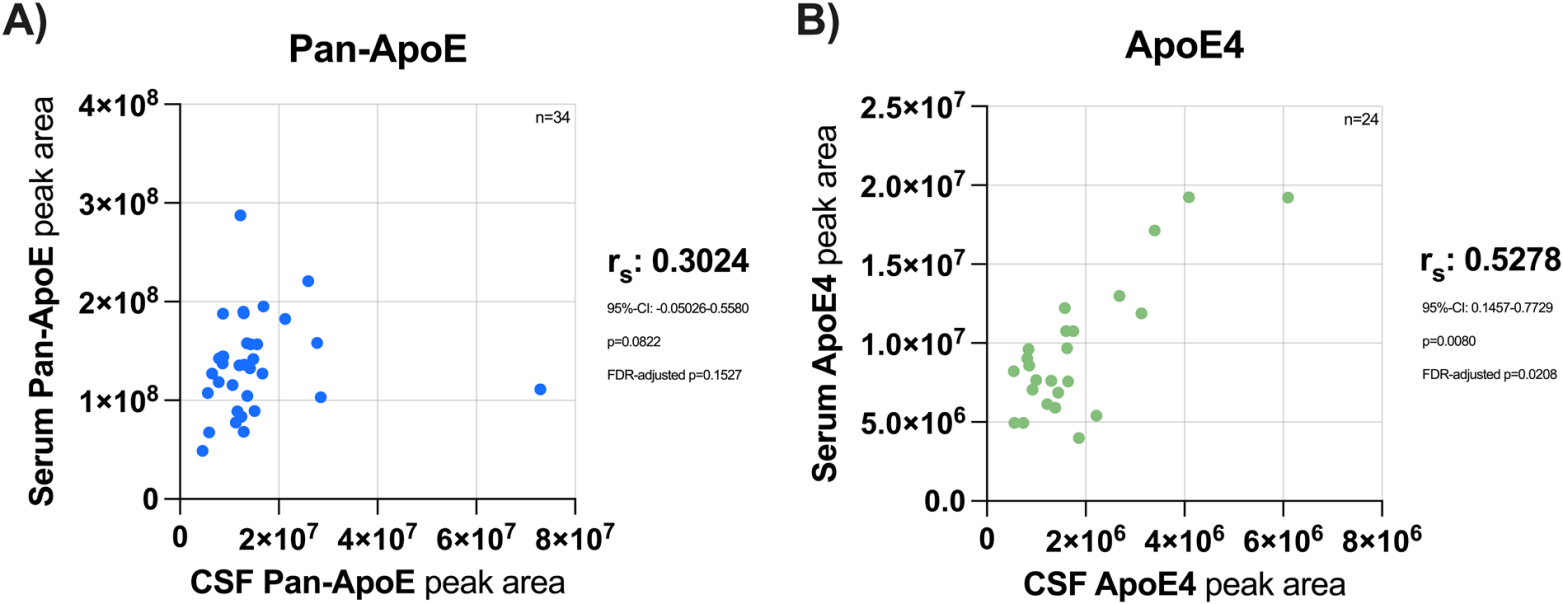
Association between CSF and serum measurements of Pan‐ApoE and ApoE4. Spearman correlation analyses were performed between dilution corrected peak area of Pan‐ApoE (*n* = 34) (A) and ApoE4 (*n* = 24) (B), as measured in cerebrospinal fluid (CSF) and serum by electrophoresis‐based capillary Western blot.

For all statistical tests, the significance level *α* was set to 0.05, with two‐sided *p*‐values.

The distribution of continuous variables was assessed using the Shapiro‐Wilk test in combination with visual inspection of histograms. Because several variables deviated from a normal distribution, nonparametric methods were used for correlation and group comparisons.

To evaluate the association between CSF and serum peak area, and CSF/serum quotients of Pan‐ApoE, ApoE4, and albumin as well as Western blot and CLEIA measurements, Spearman correlation analysis was performed. Ordinary least‐squares (OLS) linear regression was used to evaluate the relationship between the CSF/serum quotients of Pan‐ApoE or ApoE4 and the CSF/serum albumin quotient. Regression assumptions were assessed by visual inspection of residual plots and histograms of the residuals.

Mann‐Whitney tests were conducted to assess for differences between two independent groups.

A Wilcoxon signed‐rank test was performed to evaluate differences between paired Pan‐ApoE and ApoE4 CSF/serum quotients.

Samples from proteotypic ApoE4 non‐carriers with non‐detectable ApoE4 were excluded from all ApoE4‐specific analyses, including CSF/serum quotient calculations, correlation analyses, and linear regression.

To account for multiple testing, *p*‐values were adjusted using the Benjamini‐Hochberg procedure to control the false discovery rate (FDR). The correction resulted in a change in the statistical significance of the correlation between CSF Pan‐ApoE concentrations determined using the Lumipulse assay and the corresponding CSF Pan‐ApoE western blot signal (Figure 2C).

No outlier test was conducted.

## 3 Results

### 3.1 Association Between CSF and Serum Pan‐ApoE and ApoE4 Levels

CSF and serum Pan‐ApoE Western blot signals exhibited a weak, non‐significant association (*r*_s_: 0.3024, 95%‐CI: −0.05026–0.5880, *p* = 0.0822, FDR‐adjusted *p* = 0.1527), while the ApoE4 signal demonstrated a moderate correlation (*r*_*s*_: 0.5278, 95%‐CI: 0.1457–0.7729, *p* = 0.0080, FDR‐adjusted *p* = 0.0208) (Figure 3).

**FIGURE 4:**
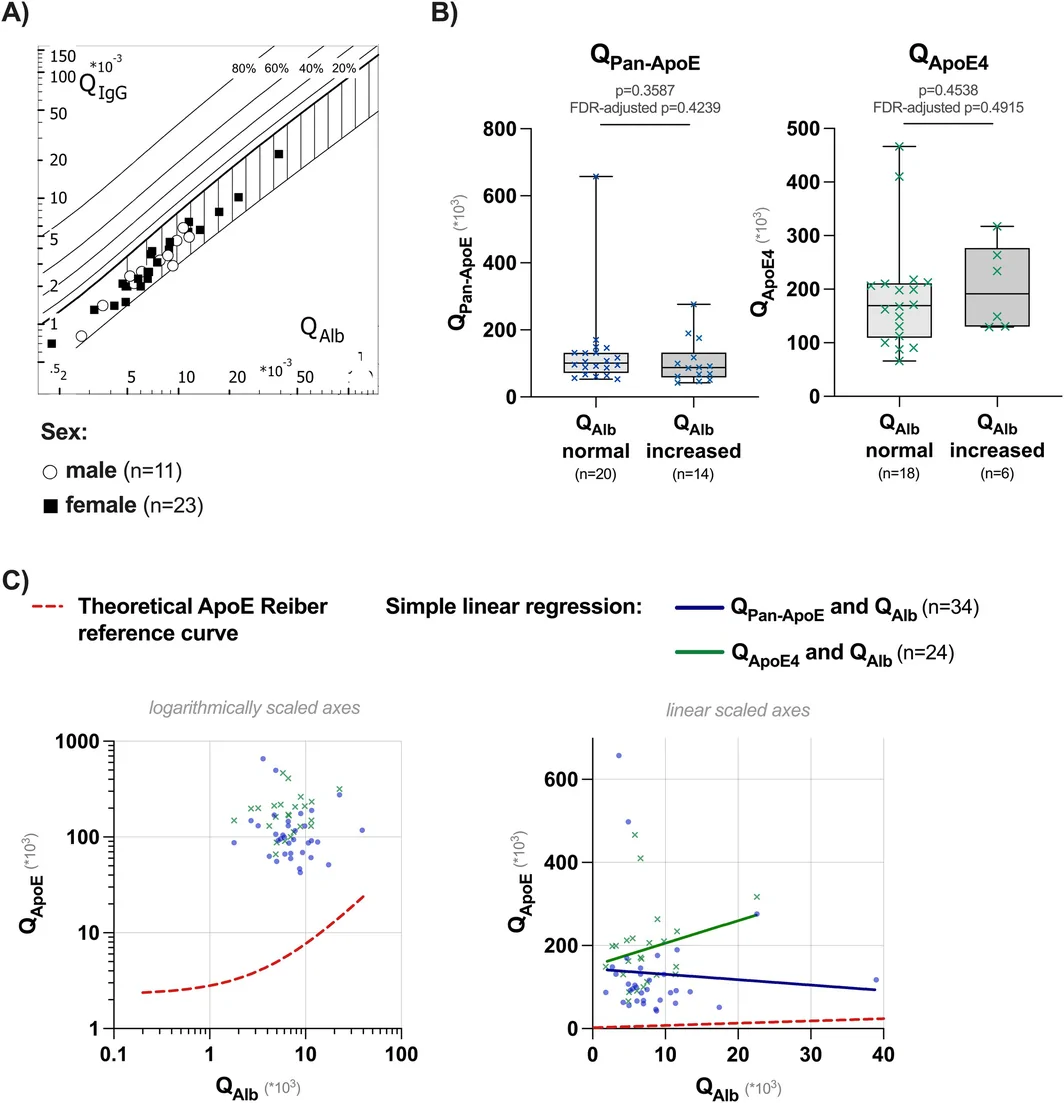
Relationship between CSF/serum quotients of Pan‐ApoE and ApoE4, albumin quotient and hydrodynamic radius. (A) Reibergram of study population (*n* = 34 participants): CSF/serum quotient of IgG (*Q*_IgG_) and albumin (*Q*_Alb_). (B) CSF/serum quotients of Pan‐ApoE (*Q*_Pan‐ApoE_) and ApoE4 (*Q*_ApoE4_) did not differ between patients with intact and dysfunctional blood‐CSF barrier, as indicated by CSF/serum albumin ratio (*Q*_Alb_). *Q*_Pan‐ApoE_: *n* = 20 probands with normal *Q*_Alb_ versus *n* = 14 with increased *Q*_Alb_ (Mann–Whitney *U* = 113, *p* = 0.3587, FDR‐adjusted *p* = 0.4239). *Q*_ApoE4_: *n* = 18 probands with normal *Q*_Alb_ versus *n* = 6 with increased *Q*_Alb_ (Mann‐Whitney *U* = 42, *p* = 0.4538, FDR‐adjusted *p* = 0.4915). Box plots show the median (center line), the interquartile range (box), and the minimum and maximum values (whiskers). Individual data points are shown as crosses. (C) The CSF/serum quotient of Pan‐ApoE (*Q*_Pan‐ApoE_, *n* = 34 probands) as well as ApoE4 (*Q*_ApoE4_, *n* = 24 probands) showed no statistically significant association with blood‐CSF barrier function. The observed mean CSF/serum quotients exceed the values expected from the approximated theoretical ApoE Reiber curve by approximately 20‐fold, which assumes CSF ApoE is solely derived from blood via passive diffusion across the blood‐CSF barrier. This model is based on IgG‐derived parameters reflecting similar hydrodynamic radius, without accounting for potential differences in ApoE isoform and lipidation. Results are shown on a double‐logarithmic scale, as commonly applied in Reiber diagrams, and additionally on a linear scale for linear regression analysis. As the theoretical ApoE Reiber curve is nearly linear within the observed range of Q_Alb_, a linear regression of the measured *Q*_Pan‐ApoE_ (*Y* = −1.296*X + 143.7, *R* ^2^: 0.0050, *p* = 0.6905, FDR‐adjusted *p* = 0.6905) as well as *Q*_ApoE4_ (*Y* = 5.435*X + 151.2, *R* ^2^: 0.0558, *p* = 0.2664, FDR‐adjusted *p* = 0.4232) and *Q*_Alb_ values was performed. CSF/serum Pan‐ApoE and ApoE4 ratio were not correlated to the blood‐CSF barrier function.

### 3.2 Dependency of Pan‐ApoE and ApoE4 CSF/Serum Quotients on Blood‐CSF Barrier Function

The albumin CSF/serum quotient (*Q*_Alb_) ranged from 1.8 to 39, with a mean *Q*_Alb_ of 8.6. Out of 34 patients, 14 had an increased Q_Alb_ (Supplementary Table 1, Figure 4A).

The relative peak area as a measure of analytes' protein concentration of Pan‐ApoE and ApoE4 was 9 and 6 times higher in serum compared to CSF, respectively. The CSF/serum quotient of Pan‐ApoE (*Q*_Pan‐ApoE_) as well as ApoE4 (*Q*_ApoE4_) did not differ statistically significant between patients with intact and dysfunctional BCSFB (Figure 4B).

The CSF/serum ApoE quotient (*Q*_Pan‐ApoE_) did not correlate statistically significantly with the CSF/serum albumin quotient (*Q*_Alb_) (*r*_s_: −0.1742, 95%‐CI: −0.4918–0.1843, *p* = 0.3244, FDR‐adjusted *p* = 0.4232, *n* = 34) (Figure 4C).

The theoretical Reiber function of ApoE reflects the estimated ApoE CSF/serum quotient as a function of *Q*_Alb_ in case all CSF ApoE would be blood‐derived via passive diffusion across the BCSFB. Comparison of *Q*_Pan‐ApoE_ with the theoretical Reiber curve revealed that the *Q*_Pan‐ApoE_ values were about 20‐fold higher than predicted by passive diffusion of ApoE across the BCSFB alone. *Q*_Pan‐ApoE_ and *Q*_Alb_ showed no linear relationship (Figure 4C). For the association between *Q*_ApoE4_ and *Q*_Alb_, analogous results were obtained regarding correlation (*r*_s_: 0.2097, 95%‐CI: −0.2237 to 0.5738, *p* = 0.3255, FDR‐adjusted *p* = 0.4232, *n* = 24) and linear relationship (Figure 4C).

### 3.3 Felgenhauer Plot: Estimation of Blood‐Derived and Intrathecal Synthesized ApoE Fraction in CSF

Comparison of the measured *Q*_Pan‐ApoE_ with the theoretical *Q*_ApoE_ proxy as a function of *Q*_Alb_ revealed that CSF ApoE levels exceeded the levels expected from passive diffusion across the BCSFB. Using the Felgenhauer plot (Figure 5), we estimated the fraction of ApoE in the CSF that originates from sources other than passive diffusion, reflecting contributions from intrathecal ApoE synthesis or potential active transport across the BCSFB.

**FIGURE 5:**
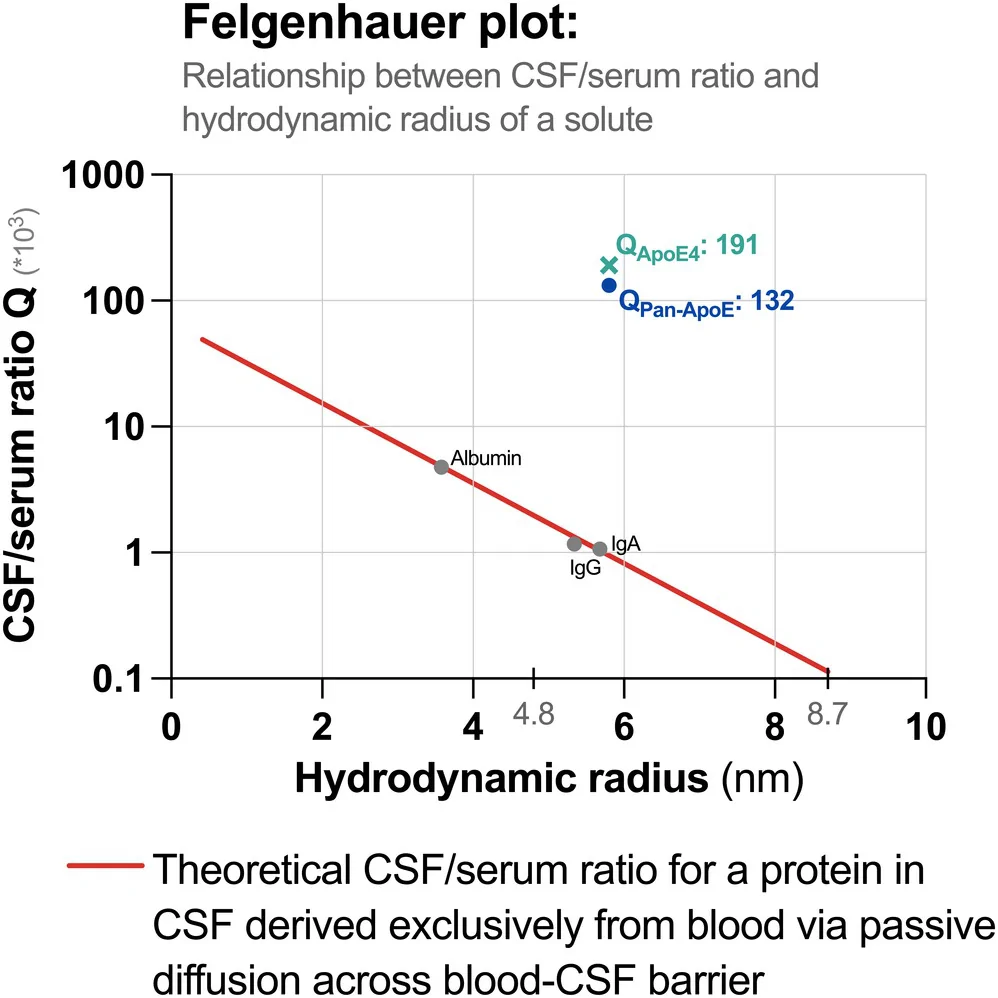
Felgenhauer plot of Pan‐ApoE and ApoE4. The Felgenhauer plot illustrates the relationship between the expected CSF/serum quotient (*Y*‐axis) and hydrodynamic radius (*X*‐axis) of a solute (e.g., protein like ApoE) assuming exclusive blood‐derived passive diffusion across the blood‐CSF barrier (red solid line, see Felgenhauer 1974). The observed mean CSF/serum quotients of Pan‐ApoE (*n* = 34) and ApoE4 (*n* = 24) are approximately 66–1320‐fold and 96–1910‐fold higher, respectively, than expected from passive diffusion alone.

The hydrodynamic radius of ApoE ranges from 4.8 to 8.7 nm, depending on isoform (ApoE4 < ApoE3 < ApoE2) and lipidation (lipidated < lipid‐free). Taking the range of the hydrodynamic radius of ApoE into account in the Felgenhauer plot, the estimated CSF/serum ratio (Q*10^3^) of ApoE would be approximately 0.1–2 depending on present ApoE isoforms and lipidation if all CSF ApoE would be derived solely from blood via passive diffusion, without any contribution from intrathecal synthesis or active transport across the BCSFB.

In contrast, using the Western blot analysis, we observed a mean *Q*_Pan‐ApoE_ of 132 (95% CI: 89.1–175.8) while the median *Q*_Pan‐ApoE_ and inter‐quartile range were 94.5 and 67.3–134.8, respectively. Based on the Felgenhauer model, passive diffusion across the BCSFB was estimated to account for approximately 0.08%–1.5% of CSF ApoE, depending on the isoform and lipidation state. The remaining 98.5%–99.92% cannot be explained by the estimated contribution of passive diffusion alone and may reflect intrathecal synthesis, active transport across the BCSFB, or a combination of both. However, these percentages represent model‐based estimates and do not allow intrathecal synthesis to be distinguished from active transport.

Mean *Q*_ApoE4_ (*10^3^) was 191 (95% CI of the mean: 155.0–231.9, median: 169.5, IQR: 129.6–216.3) measured by the Western blot. Q_Pan‐ApoE_ and Q_ApoE4_ differed between paired measurements (*p* < 0.0001, FDR‐adjusted *p* = 0.0013, *n* = 24 pairs), indicating that even a smaller fraction of ApoE4 passively diffuses across the BCSFB into the CSF.

To complement the Western blot analysis, CSF/serum quotients for Pan‐ApoE and ApoE4 were calculated based on the absolute concentrations obtained from Lumipulse measurements in samples for which both CSF and serum were available. Based on the Lumipulse measurements, the mean *Q*_Pan‐ApoE_ and *Q*_ApoE4_ were 72 (*n* = 31) and 93 (*n* = 24), respectively. This corresponds to an estimated CSF Pan‐ApoE fraction of 0.2%–2.2% attributable to passive diffusion across the BCSFB, based on the Felgenhauer model.

## 4 Discussion

To monitor the effects elicited by future therapies targeting ApoE, it is crucial to understand the compartmentalization of ApoE between the CNS and the periphery, given that brain‐derived and peripheral blood ApoE may represent distinct pools (Kraft et al. 1989; Linton et al. 1991).

Following Reiber's model, which distinguishes passive diffusion across the BCSFB and intrathecal production of CSF proteins, our data estimate that only a minor fraction (0.08%–1.5%) of CSF ApoE can be explained by passive diffusion from the peripheral blood, depending on the assumed hydrodynamic properties of the different ApoE isoforms and their extent of lipidation (Hubin et al. 2019). The remaining fraction (98.5%–99.92%) may reflect intrathecal ApoE synthesis, active transport of ApoE across the BCSFB, or a combination of both. Active transport may also contribute to the substantially higher CSF/serum ApoE quotient than would be expected from passive diffusion alone, a mechanism not accounted for in Reiber's classical model. Active transport mechanisms have been described for trace and essential elements at the BCSFB. Several potential ApoE receptor candidates (e.g., LRP1, LDLR, VLDLR, megalin/LRP2) are expressed in both the central nervous system and peripheral tissues (Holtzman et al. 2012; Spuch and Navarro 2010; Zhao et al. 2018). Their expression at the human BCSFB remains poorly characterized. In mice, human ApoE did not cross the BCSFB, despite demonstrated interaction with the murine ApoE receptors (Liu et al. 2012). Following liver transplantation, recipients adopt the donor's ApoE phenotype in the peripheral blood, reflecting the liver as the primary source of peripheral ApoE (Fernández‐Miranda et al. 1997; Kraft et al. 1989; Linton et al. 1991). In contrast, the ApoE phenotype in CSF remains unchanged, providing strong evidence that ApoE pools in the periphery and the CNS are largely compartmentalized with minimal exchange (Fernández‐Miranda et al. 1997; Kraft et al. 1989; Linton et al. 1991). We hypothesize that if there were substantial active transport of peripheral blood ApoE across the BCSFB, both the recipient's and donor's ApoE isoforms or phenotype would be detectable in the CSF, which was not the case in human studies (Linton et al. 1991). These findings are compatible with the hypothesis of a predominantly intrathecal origin of CSF ApoE, while not excluding a contribution from active transport of ApoE across the BCSFB or a combination of both mechanisms (Reiber 1994, 2016).

To our knowledge, this is the first study to determine CSF/serum quotients for Pan‐ApoE and ApoE4 and estimate the fraction attributable to passive diffusion across the BCSFB by analyzing paired CSF and serum samples using an electrophoresis‐based capillary Western blot. Some previous estimates of the intrathecal fraction of ApoE were based on published mean CSF and serum concentrations from different studies using different methods (e.g., ELISAs), implying methodological heterogeneity across the applied measurement techniques. Consequently, analyzed CSF and serum samples were not necessarily obtained as paired samples from the same patients.

As a note in a book chapter more than 25 years ago, an estimated ApoE CSF/serum quotient (*Q*_ApoE_*10^3^) of 50–63 has been reported based on the molecular weight of ApoE (34 kDa), corresponding to a CSF ApoE fraction of 90% that is intrathecally synthesized without taking into account potential active ApoE transport across the BCSFB (Thomas 1998; Pitas et al. 1987). This assumption was based on independent studies using different methods. Additionally, for estimating the protein concentration filtrated at the BCSFB, the hydrodynamic radius is more accurate than the molecular weight (Felgenhauer 1974). ApoE has a relatively large hydrodynamic radius for its size, which is comparable to those of IgG or IgA depending on the lipidation and isoform of ApoE (Hubin et al. 2019; Raulin et al. 2019; Reiber 1994). Therefore, the CSF/serum ApoE quotients estimated based on the molecular weight of ApoE could have led to an overestimation of the blood‐derived ApoE fraction and therefore to an underestimation of the intrathecally synthesized or actively transported ApoE fraction (Felgenhauer 1974; Hubin et al. 2019). In contrast, another ELISA study analyzing paired CSF/serum samples reports that the serum ApoE concentration is 12 times higher than those in CSF (Carlsson et al. 1991). Our findings for Pan‐ApoE showed a similar pattern, with serum concentrations approximately ninefold higher than CSF concentrations. In addition, we separately quantified ApoE4 and observed serum concentrations approximately six fold higher than those in CSF.

The resulting CSF/serum quotients were subsequently compared with the theoretical values predicted by the Felgenhauer model to estimate the fraction of CSF ApoE attributable to passive diffusion across the BCSFB, as previously done for proteins like α‐Synuclein, Progranulin and Erythropoietin (Deisenhammer et al. 2015; Feneberg et al. 2016; Oeckl et al. 2016; Widl et al. 2007). In our study, the estimated fraction of CSF ApoE attributable to passive diffusion across the BCSFB ranges from 0.08% to 1.5%, while the remaining fraction (98.5%–99.92%) may be intrathecally synthesized, actively transported across the BCSFB, or both. These values should be interpreted as model‐based estimates, as they depend on assumptions regarding the hydrodynamic properties of ApoE, including isoform and extent of lipidation, which are the following. Firstly, the hydrodynamic radius of ApoE differs between the isoforms, with a tendency for ApoE2 to have the largest, followed by ApoE3, and ApoE4 the smallest (Hubin et al. 2019; Raulin et al. 2019). Secondly, the lipidation of ApoE affects its hydrodynamic radius, with lipid‐free ApoE exhibiting a larger hydrodynamic radius than lipid‐bound or lipidated ApoE, likely due to the greater tendency of lipid‐free ApoE to aggregate (Hubin et al. 2019). Several studies report that nearly all ApoE is lipid‐bound in blood, therefore the smaller hydrodynamic radius of lipid‐bound ApoE should be considered (Castro and Fielding 1984). Thirdly, we evaluated patients with different *APOE* genotypes (2 *ε4*/*ε4*, 21 *ε3*/*ε4*, 3 *ε3*/*ε3*, 1 *ε2*/*ε3*, 7 with unknown *APOE* genotype). Among the 27 patients with available *APOE* genotyping, 23 were *APOE ε4* carriers. One additional patient without available genotype data showed detectable ApoE4 by the isoform‐specific antibody, although this does not allow determination of whether the patient was heterozygous or homozygous for *APOE ε4*. The anti‐Pan‐ApoE antibody used may exhibit different affinities for each isoform, meaning that the measured signal reflects a combination of the present isoform levels and the antibodies binding preferences (Martínez‐Morillo et al. 2014). Moreover, the contribution of isoform expression to Pan‐ApoE levels differ between CSF and plasma, for example, for the *APOE ε3/ε4* genotype (Martínez‐Morillo et al. 2014). Therefore, differences in isoform composition between CSF and peripheral blood may affect the comparability of Pan‐ApoE signal intensities between the two matrices. Consequently, we cannot derive information regarding the relative proportions of individual isoforms from a sample.

The higher CSF/serum ratio of ApoE4 compared with Pan‐ApoE would not be expected based on the Felgenhauer plot, given the smaller hydrodynamic radius of the ApoE4 isoform compared with the mean hydrodynamic radius of the ApoE2, ApoE3, and ApoE4 isoforms collectively detected by the anti‐Pan‐ApoE antibody. These data could be explained by ApoE isoform‐specific differences in catabolism: in peripheral blood, ApoE4 exhibits a faster turnover, followed by ApoE3, whereas ApoE2 displays the longest half‐life. However, such isoform‐specific ApoE kinetics have not been observed in CSF (Wildsmith et al. 2012). This could be a consequence of differential sialylation of the distinct ApoE pools, as sialylation has been shown to protect proteins from degradation and confer stability (Russell et al. 2009; Sarkar and Wintrode 2011). While only 10%–20% of peripheral blood ApoE is sialylated, 60%–70% of CNS ApoE is glycosylated (Hu et al. 2020). Glycosylation levels are also influenced by the ApoE isoform, with ApoE4 showing the lowest and ApoE2 the highest proportion of glycosylated species (Hu et al. 2020). Therefore, the minor fraction of CSF ApoE originating from peripheral blood may be further reduced in the CSF due to its lower sialylation compared to CNS‐derived ApoE. A potential isoform‐, lipidation‐ or glycosylation‐dependent active transport of ApoE across the BCSFB could also contribute to differences in CSF concentrations of ApoE isoforms.

CSF ApoE levels and therefore CSF/serum ApoE quotients might also be influenced by the extent of glymphatic transport of ApoE via convective bulk flow into the brain parenchymal interstitial fluid, which is mediated by aquaporin‐4. This glymphatic transport follows an ApoE‐isoform specific (ApoE2 > ApoE3 > ApoE4) radial distribution pattern around brain parenchymal penetrating arterial vessels and is dysfunctional in the presence of sleep deprivation in mice (Achariyar et al. 2016).

CSF protein concentration gradients may exist between ventricular and lumbar CSF, depending on whether the proteins are brain‐derived or synthesized by the leptomeninges and on their absorption along the subarachnoid space (Deisenhammer et al. 2015). Previous studies have shown that mean ApoE concentrations are slightly higher in ventricular CSF compared to lumbar CSF, with a difference of up to 5 μg/mL, while the mean lumbar ApoE CSF concentration is approximately 9–13 μg/mL (Kay 2003; Wahrle et al. 2007). This decreasing rostro‐caudal gradient is typical for brain‐derived CSF proteins (Reiber 2001). In the present study, only lumbar CSF samples were analyzed. Therefore, the observed CSF/serum ApoE quotients may be marginally influenced by the lumbar site of CSF collection. However, as lumbar puncture is the most commonly used CSF sampling method, our results are comparable with those of most published studies (Carlsson et al. 1991; Kay 2003; Pitas et al. 1987).

The theoretical Reiber curve for ApoE represents an estimation, as the parameters were adopted from IgG due to the comparable hydrodynamic radius of ApoE and IgG, which is an important determinant of passive diffusion across the BCSFB (Hubin et al. 2019; Reiber 1994, 2001). This approximation has been applied for other proteins in previous studies, e.g., Mannan‐binding lectin or C4 complement protein to estimate the intrathecal fraction (Padilla‐Docal et al. 2009; Reiber et al. 2012). Reiber curves for IgG, IgA, and IgM are based on empirical data from over 4000 patients, providing a robust basis for empirical analysis (Reiber 1994). In the present study, evaluation of the 34 patients showed that the observed mean CSF/serum Pan‐ApoE quotient was about 20‐fold higher than predicted by the theoretical ApoE Reiber curve. This model provides an approximation of the expected CSF/serum quotient for passive diffusion across the BCSFB, using the CSF/serum albumin quotient as an indicator of BCSFB function and Reiber parameters (a, b, c) derived from IgG due to a similar hydrodynamic radius to ApoE (Hubin et al. 2019; Reiber 1994). However, it does not account for potential isoform‐ and lipidation‐dependent differences in the hydrodynamic properties of ApoE, which may influence its passive diffusion across the BCSFB, nor for potential receptor‐mediated transport across the BCSFB or receptor interactions within the CNS (Holtzman et al. 2012; Hu et al. 2020; Martínez‐Morillo et al. 2014; Wildsmith et al. 2012). Nevertheless, this supports the conclusion that the majority of CSF ApoE cannot be explained by passive diffusion across the BCSFB alone and may therefore reflect intrathecal ApoE synthesis, active transport across the BCSFB, or a combination of both.

In our study, no statistically significant association between CSF/serum ApoE quotients and BCSFB function was observed, which is typical for a brain‐derived CSF protein (Deisenhammer et al. 2015; Oeckl et al. 2016; Reiber 1994, 2016). Given the relatively small sample size, this finding should be interpreted with caution, but is consistent with a previous study in a larger cohort, which found no linear relationship or correlation between CSF ApoE concentration and *Q*_Alb_ (Carlsson et al. 1991).

ApoE4 levels in paired serum and CSF samples were correlated statistically significant, while Pan‐ApoE were not. The ApoE4 result stands in contrast with previous studies, which reported no correlation between CSF and plasma or serum ApoE concentrations (Fagan et al. 2000; Fukumoto et al. 2003; Yamauchi et al. 1999) or just low regression coefficients (Cruchaga et al. 2012). Potential reasons for these discrepancies include the relatively small sample size in our study, methodological differences, and the influence of the *APOE* genotype on protein expression (Begcevic Brkovic et al. 2022; Cruchaga et al. 2012). Previous studies used ELISA, whereas we employed a novel electrophoresis‐based Western blot to detect Pan‐ApoE as well as ApoE4 levels. The observed correlation between CSF and serum ApoE4 levels may in part be driven by the *APOE* genotype itself, which influences ApoE expression in both the central nervous system and peripheral tissues, with individuals carrying the *APOE ε4*/*ε4* genotype exhibiting the lowest ApoE concentrations and those carrying the *APOE ε2*/*ε2* genotype the highest (Begcevic Brkovic et al. 2022; Cruchaga et al. 2012).

Another limitation is that circadian variation and volume of sampled CSF are potential confounders that have not been evaluated regarding the CSF/serum ApoE quotient (Deisenhammer et al. 2015; Wildemann et al. 2010). Pan‐ApoE concentrations in CSF and serum are known to be increasing with age, but this effect on CSF/serum quotients has not been evaluated yet (Baker‐Nigh et al. 2016; Kaneva et al. 2013; Schiele et al. 2000). However, we did not observe any association between CSF sample volume or age and the CSF/serum quotients of either Pan‐ApoE or ApoE4. Previous studies reported disease‐related differences in CSF ApoE concentrations, but whether CSF/serum ApoE or ApoE4 quotients differ between neurological diseases has, to our knowledge after extensive literature research, not been systematically investigated (Baker‐Nigh et al. 2016; Carlsson et al. 1991; Cruchaga et al. 2012; Talwar et al. 2016). To evaluate the association between CSF ApoE and a broad range of Q_Alb_ values, we therefore included patients with different underlying neurological diseases in our cohort (Supplementary Table 2). Future studies in larger disease‐specific cohorts are needed to determine potential disease‐related effects, including the influence of AD on ApoE CSF/serum quotients, given the association between AD and the *APOE ε4* genotype (Williams et al. 2026).

The use of serum as a sample matrix may potentially affect the recovery of ApoE‐containing, particularly lipidated, particles, as coagulation could result in the association of ApoE species with the fibrin clot (Talens et al. 2012). This effect may differ between ApoE isoforms, given their differential association with lipid particles (Blanchard et al. 2018; Nguyen et al. 2010). Consequently, the ApoE composition measured in serum may not fully reflect the in vivo distribution of ApoE species. However, in a paired analysis of plasma and serum samples using the Lumipulse assay, mean ApoE concentrations differed only minimally between the two matrices, arguing against a major systematic effect of serum as the sample matrix (Kortmann et al. 2026). Nevertheless, selective effects on specific lipidated ApoE species in our Western blot cannot be excluded and could lead to an overestimation of the CSF/serum quotient and, consequently, an underestimation of the contribution of passive diffusion to the CSF ApoE pool. Moreover, the sample diluent used in this study contains 6% CHAPS and 10% SDS, which may affect the lipidation and conformation of ApoE‐containing particles (Wang et al. 1996; Zhuo and Yuan 2020). The use of different detergents could result in different outcomes due to detergent‐dependent effects on particle lipidation.

Despite the fact that our Western blot analysis reports arbitrary units (peak area under the curve), dilution‐corrected CSF/serum quotients can be calculated from these relative signal intensities without requiring absolute protein concentrations. Although these values should be considered estimates, as matrix comparability may be limited by differences in antibody specificity, including differential binding to ApoE isoforms and differences in glycosylation. Of note, capillary electrophoresis‐based Western blot platforms have been successfully used for the quantification of various proteins in biological samples (Beekman et al. 2018; Erdmann et al. 2024; Lu et al. 2018; Mankowski et al. 2023; Myskiw et al. 2024). This further supports the validity of using this technique as a comparative tool for protein quantification in CSF and serum.

Notably, to our knowledge, this is the first study to quantify CSF Pan‐ApoE and ApoE4 using the Lumipulse ApoE assays after an extensive review of the literature. We observed a moderate (*r*_s_ > 0.5) correlation between Western blot signal intensities and the absolute concentrations of serum Pan‐ApoE and ApoE4, as well as CSF ApoE4, measured by Lumipulse CLEIA. CSF Pan‐ApoE levels were not significantly correlated between Western blot and Lumipulse measurements after correction for multiple testing, possibly due to the limited sample size. Other potential explanations include the fact that serum and CSF samples were analyzed independently using different calibrations, as well as different antibodies in the Western blot and Lumipulse assays. Because ApoE levels in CSF are approximately 10 times lower than those in serum, quantification of ApoE in CSF using the Lumipulse Pan‐ApoE and ApoE4 assays may be problematic. The Lumipulse assays report concentrations in increments of 0.1 μg/mL, which may limit the resolution of measurements at the low concentrations typically encountered in CSF and make small differences in ApoE levels difficult to distinguish (Hesse et al. 2000; Minta et al. 2020; Wahrle et al. 2007). The lower limits of linearity of the Lumipulse Pan‐ApoE assay established for peripheral blood may fall below the typical concentrations of ApoE found in CSF samples (Degrieck et al. 2023; Hesse et al. 2000; Minta et al. 2020; Wahrle et al. 2007). However, in every tested CSF sample, we measured a Pan‐ApoE concentration above the previously reported limits of quantification (Degrieck et al. 2023). Therefore, we calculated CSF/serum quotients based on the Lumipulse measurements in addition to the Western blot results, corresponding to a CSF Pan‐ApoE fraction transported across the BCSFB via passive diffusion of 0.2%–2.2%. This is consistent with the 0.08%–1.5% fraction estimated from the Western blot measurements, with both estimates falling within the same order of magnitude. The slight discrepancy may be attributable to differences in the antibodies used, assay calibration, inter‐assay variability in the Lumipulse measurements, and concentrations close to the limit of quantification of the Lumipulse assays, as discussed above.

To summarize, by using paired CSF and serum samples analyzed by electrophoresis‐based capillary Western blot, we estimate that only a minority (< 1.5%) of CSF ApoE originates from peripheral blood via passive diffusion across the BCSFB. The remaining proportion of CSF ApoE may be actively transported across the BCSFB, synthesized intrathecally, or both. Comparable findings were observed for the ApoE4 isoform by Western blot and when analyzing Pan‐ApoE and ApoE4 quotients using the Lumipulse assays. The present findings and previous studies are consistent with the hypothesis of a predominantly intrathecal origin of CSF ApoE, based on the lack of a statistically significant association between the CSF/serum ApoE quotient and BCSFB function, the decreasing rostrocaudal CSF concentration gradient of ApoE, experimental data demonstrating no detectable passage of human ApoE across the murine BCSFB despite its interaction with murine ApoE receptors, and the preservation of the recipient ApoE phenotype in CSF after liver transplantation despite a change in the peripheral ApoE phenotype. However, these values should be interpreted as model‐based estimates, as they depend on assumptions regarding the hydrodynamic properties of ApoE, including isoform and degree of lipidation. Further studies are required to elucidate the contribution of active ApoE transport across the BCSFB and intrathecal synthesis to CSF ApoE levels. This will be important for the development of future ApoE‐targeting therapeutic strategies, such as experimental *APOE* allele switching or replacement approaches, by clarifying the relative contribution and accessibility of peripheral and centrally synthesized ApoE pools.

## 5 Conclusion

Our model‐based findings estimate that less than 1.5% of CSF ApoE originates from peripheral blood via passive diffusion across the BCSFB. However, the contribution of intrathecal ApoE synthesis and active transport across the BCSFB remains to be elucidated.

## Author Contributions

**Eric Kortmann:** conceptualization, investigation, writing – original draft, methodology, visualization, formal analysis, project administration, data curation. **Petra Steinacker:** conceptualization, investigation, methodology, writing – review and editing, supervision, project administration, formal analysis. **Arthur Guthknecht:** writing – review and editing. **Lorenzo Barba:** writing – review and editing. **Markus Otto:** conceptualization, funding acquisition, investigation, writing – review and editing, methodology, project administration, supervision, resources, formal analysis.

## Funding

MO received research support from Horizon Europe Health: PredictFTD, SYNAPSING (101156175, 101156566), ERA‐NET NEURON (MOODMARKER) (01EW2008), and the German Federal Ministry of Education and Research (BMBF) (FTLDc 01GI1007A).

## Ethics Statement

The study was approved by Martin Luther University's Halle‐Wittenberg Ethics Committee (2021‐101) and conducted according to the latest version of the Declaration of Helsinki. All participants gave informed consent to participate in the study before taking part.

## Consent

All participants gave written informed consent to participate in the study.

## Conflicts of Interest

The authors declare no conflicts of interest.

## Supporting information


**Table 1.** Patient characteristics. *Q_Alb_: CSF/serum albumin quotient. † SD: standard deviation. Description of data: Patients were categorized into three diagnostic groups: inflammatory neurological diseases, neurodegenerative diseases, and psychiatric diseases. Age, sex, *APOE* genotype, CSF, and serum albumin concentrations, and CSF/serum albumin quotients are reported for each group as well as for all enrolled patients.
**Table 2:** Patient diagnosis. Description of data: The Supplementary Table contains detailed information on patient diagnosis, age, sex, blood‐CSF barrier function, and *APOE ε4* status of the enrolled patients.

## Data Availability

The data that support the findings of this study are available from the corresponding author upon reasonable request.
