# Supplementary material for: Limited Passive Diffusion of Apolipoprotein E Across the Human Blood–CSF Barrier: Evidence From Paired CSF/Serum Quotients
Source: J Neurochem. 2026 Nov 9;170(11):e70585. doi: 10.1111/jnc.70585 (PMC13746799; doi:10.1111/jnc.70585)

Supplementary Material:

Title: Limited passive diffusion of apolipoprotein E across the human blood-CSF barrier: Evidence from paired CSF/serum quotients

Authors: Eric Kortmann[a,b], Petra Steinacker[a,b], Arthur Guthknecht[a,b], Lorenzo Barba[a,b], Markus Otto[a,b]*

Author affiliations:

[a]Department of Neurology, University Hospital Halle (Saale), 06120 Halle (Saale), Germany

[b]Martin Luther University Halle-Wittenberg, Medical Faculty, 06112 Halle (Saale), Germany

Supplementary Material: Table 1

- Title of data: “Table 1: Patient characteristics. *QAlb: CSF/serum albumin quotient. † SD: standard deviation.”

- Description of data: Patients were categorized into three diagnostic groups: inflammatory neurological diseases, neurodegenerative diseases, and psychiatric diseases. Age, sex, *APOE* genotype, CSF and serum albumin concentrations, and CSF/serum albumin quotients are reported for each group as well as for all enrolled patients.

Table 1: Patient characteristics

	**Number of patients with blood-CSF barrier dysfunction** (Q_{Alb}^{*} > age-specific cut-off)	**Mean Age** (SD †) in years	**Male sex** n (%)	***APOE ε4* homozygous genotype**	***APOE ε4* heterozygous genotype**	**Non-*APOE ε4* genotype**	**Unknown *APOE* genotype**	**CSF total protein concentration** mg/L	**Mean CSF albumin concentration** (SD†) mg/L	**Mean serum albumin concentration** (SD†) g/L	**Mean CSF/serum albumin quotient** (Q_{Alb}^{*} *10^{3})
**All**	**14/34 (41.7%)**	**65.6 (16.5)**	**11/34 (32.4%)**	**2/34 (5.9%)**	**21/34 (64.7%)**	**4/34 (11.8%)**	**7/34 (20.6%)**	**531.5 (351)**	**336.1 (252)**	**39.3 (3.9)**	**8.6 (6.7)**
**Diagnosis group**											
**Inflammatory neurological diseases**	7/8 (88%)	50 (17.8)	3/8 (37.5%)	0	1 *ε3/ε4* (4.7%)	0	7	785.9 (598.2)	515 (436.9)	37.6 (2.9)	13.5 (11.7)
**Neurodegenerative diseases**	7/24 (28%)	72.6 (9.5)	8/24 (33.3%)	2/24 (8%)	18 *ε3/ε4* (85.7%)	3 *ε3/ε3* (75%) 1 *ε2/ε3* (25%)	0	464.6 (185.4)	284,98 (146)	40.2 (4.2)	7.45 (3.6)
**Psychiatric diseases**	0/2	42 (21.2)	0/2	0	2 *ε3/ε4* (9.5%)	0	0	317.8 (167.1)	173,9 (107.8)	38.8 (1.4)	4.4 (2.5)

$^{*}Q_{Alb}$: CSF/serum albumin quotient. †SD: standard deviation.

<u>Supplementary Material: Table 2</u>

- Title of data: “Table 2: Patient diagnosis”

- Description of data: The Supplementary Table contains detailed information on patient diagnosis, age, sex, blood-CSF barrier function, and *APOE ε4* status of the enrolled patients.

<u>Table 2</u>: Patient diagnosis

**Diagnosis**	**Age** (SD)	**Male sex** (%)	**Number of participants** (in brackets: number of those with blood-CSF barrier dysfunction: Q_{Alb} > age-specific cut-off)	***APOE ε4*** carrier, *n* (%)
**All/Total**	**65.6 (16.5)**	**11/34 (32.4%)**	**34 (14)**	**23/34 (67.6%)**
Guillan-Barrè syndrome (GBS)*	/		5 (5)	unknown
Bickerstaff brainstem encephalitis*			1 (1)	unknown
Facial and hypoglossal nerve paresis and CSF pleocytosis of unknown etiology*			1 (1)	unknown
Alzheimer's disease†			18 (4)	18/18 (100%)
Alzheimer's disease A+T+N+			16 (5)	16/16 (100%)
Alzheimer's disease A+T-N+			1 (0)	1/1(100%)
Alzheimer's disease A+T-N-			1 (1)	1/1 (100%)
Vascular dementia†			3 (1)	1/3 (33.3%)
Behavioural variant			1 (0)	0

frontotemporal dementia (bvFTD)†			
Spinocerebellar ataxia (SCA)†		2 (0)	1/2 (50%)
Immune-mediated necrotizing myopathy (IMNM)*		1 (0)	1/1 (100%)
Depression-related cognitive impairment‡		1 (0)	1/1 (100%)
Psychogenic nonepileptic seizures (PNES)‡		1 (0)	1/1 (100%)

Diagnosis group in main text: *: Inflammatory neurological diseases. †: Neurodegenerative diseases. ‡: Psychiatric diseases.

Supplementary Material: Western Blots of data

- Title of data: “Pan-ApoE and ApoE4 Western Blots of CSF and serum”

- Description of data: Western Blots of CSF (diluted to 25 mg/L total protein concentration) and serum (1:400 diluted) probed with 1:10 diluted primary antibodies anti-Pan-ApoE (RRID:AB_2878525, cat. no. 18254-1-AP, proteintech, host: rabbit) and anti-ApoE4 (no RRID found, cat. no. 39327, CellSignaling, host: rabbit) and a 1:20 diluted HRP-conjugated goat anti-rabbit-IgG (RRID:AB_2534782, cat. no. A16110, invitrogen) as secondary antibody. See method section for detailed description of capillary Western blot. Abbreviations in figures: 18254-1-AP: anti-Pan-ApoE antibody. E5M4L: anti-ApoE4 antibody. IVG: anti-rabbit-IgG secondary antibody. Liq: cerebrospinal fluid. Ser: serum.

**Western Blot:
anti-Pan-ApoE**

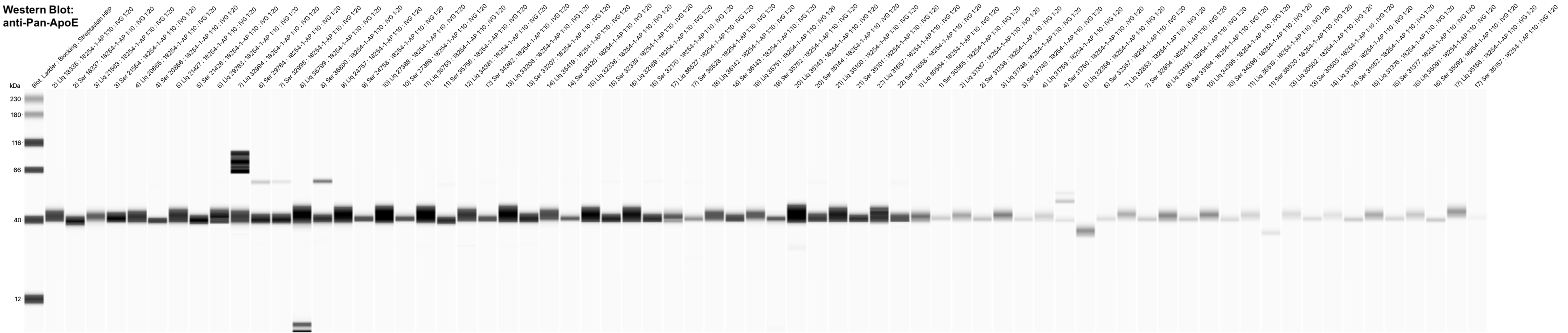

Western Blot:
anti-ApoE4

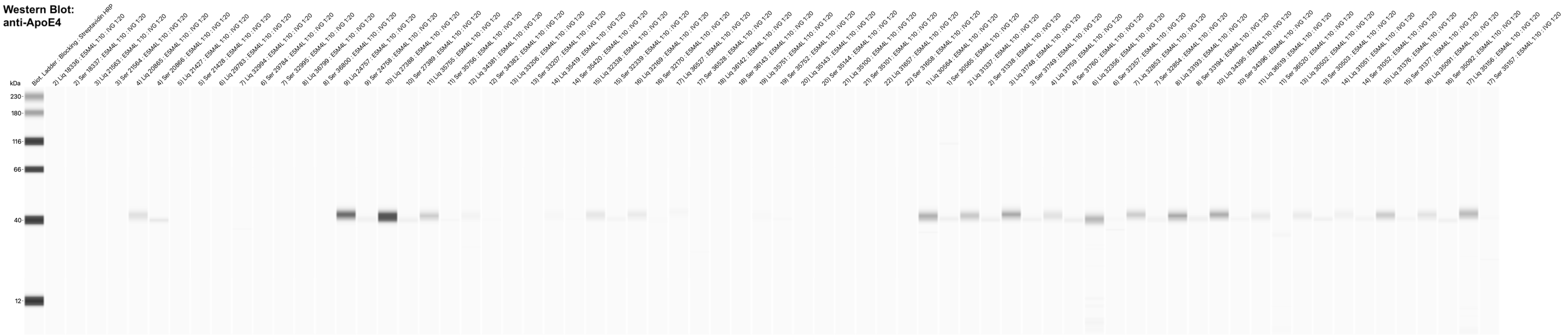

Supplement: Supplementary file 1 [file JNC-170-e70585-s001.pdf]
